# Shear-Regulated Extracellular Microenvironments and Endothelial Cell Surface Integrin Receptors Intertwine in Atherosclerosis

**DOI:** 10.3389/fcell.2021.640781

**Published:** 2021-04-06

**Authors:** Fan-E Mo

**Affiliations:** ^1^Department of Cell Biology and Anatomy, College of Medicine, National Cheng Kung University, Tainan, Taiwan; ^2^Institute of Basic Medical Sciences, College of Medicine, National Cheng Kung University, Tainan, Taiwan

**Keywords:** disturbed flow atherosclerosis, extracellular matrix, integrin, matricellular proteins, endothelial dysfunction, atheroprotection

## Abstract

Mechanical forces imposed by blood flow shear stress directly modulate endothelial gene expression and functional phenotype. The production of extracellular matrix proteins and corresponding cell-surface integrin receptors in arterial endothelial cells is intricately regulated by blood flow patterns. Laminar blood flow promotes mature and atheroresistant endothelial phenotype, while disturbed flow induces dysfunctional and atheroprone endothelial responses. Here, we discuss how hemodynamic changes orchestrate the remodeling of extracellular microenvironments and the expression profile of the integrin receptors in endothelial cells leading to oxidative stress and inflammation. Targeting the interaction between matrix proteins and their corresponding integrins is a potential therapeutic approach for atherosclerosis.

## Introduction

**Figure 1:**
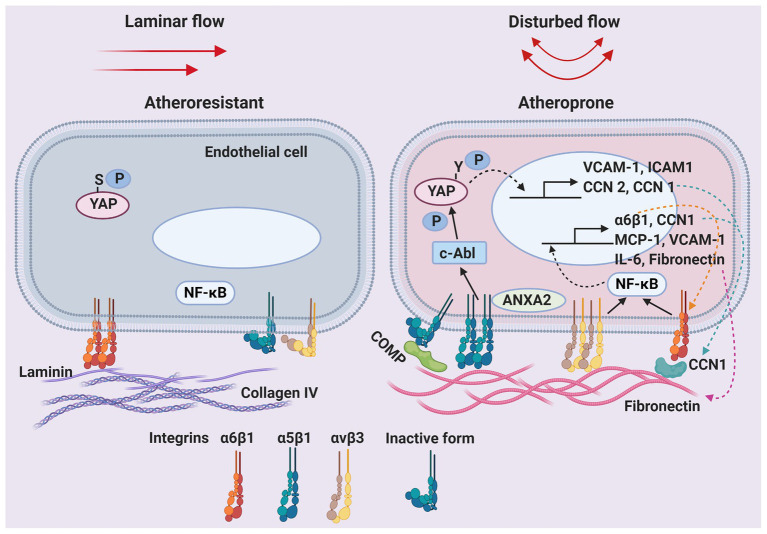
Shear-regulated extracellular microenvironments and endothelial integrin receptors in atherosclerosis. Schematic of the dynamic changes in the composition of the endothelial basement membrane and cell-surface integrin receptors by unidirectional laminar blood flow or oscillatory disturbed flow. Solid arrows: activation of downstream effectors. Dashed arrows: translocation of proteins. ANXA2, annexin A2; COMP, cartilage oligomeric matrix protein; ICAM1, intercellular adhesion molecule 1; IL-6, interleukin-6; MCP-1, monocyte chemoattractant protein 1; NF-κB, nuclear factor-κB; P, phosphorylation; S, serine; VCAM-1, vascular cell adhesion molecule 1; Y, tyrosine; YAP, yes-associated protein.

Though risk factors, such as hyperlipidemia, hypertension, and hyperglycemia, pose a threat to the entire arterial system, atherosclerosis preferentially occurs at arterial branches or curvatures, where the local blood flow is disturbed (Chiu and Chien, 2011). Blood flow imposes fictional drag, called sheer stress, directly to the inner lining endothelial cells (ECs). Laminar blood flow found at the straight part of artery generates unidirectional shear stress and promotes functional endothelial phenotype (atheroprotective). By contrast, disturbed flow generates low and oscillatory sheer stress, and induces EC activation and maladaptive alterations in endothelial functional phenotype (atheroprone; Hsu et al., 2019). Low endothelial shear stress correlates with increased plaque burden and risks of rupture in patients with coronary artery disease (Chatzizisis et al., 2008; Stone et al., 2012). Endothelial dysfunction by disturbed flow is manifested by the lack of nitric oxide production, and chronic inflammatory response mediated through the pleiotropic transcription factor nuclear factor-κB (NF-κB; Gimbrone and Garcia-Cardena, 2016). These atheroprone genes include cell-surface adhesion molecules (such as vascular cell adhesion molecule-1; Korenaga et al., 1997), secreted cytokines (such as interleukin-1 and monocyte chemoattractant protein 1; Hsu et al., 2019), and prothrombotic mediators (such as von Willebrand factor; Zhu et al., 2020). Recent studies using systems biology approaches have demonstrated that low endothelial shear stress causes aberrant reactivation of vascular developmental signaling pathways, such as BMP-TGFβ, WNT, Notch, HIF1α, TWIST1, and HOX family genes, leading to increased inflammation and vascular permeability (Souilhol et al., 2020). Consequently, the infiltration of monocytes/macrophages rises, the uptake of lipid elevates, local inflammation and oxidative stress intensifies, and the number of vascular smooth muscle cells increases in the intima, leading to the formation of atherosclerotic plaques. Emerging evidence suggests that local microenvironment also plays a major role in regulating endothelial cell function and regional susceptibility to atherosclerosis (Yurdagul et al., 2016). Hemodynamics may affect endothelial remodeling and change subendothelial matrix composition. Concurrently, the expression profile of cell-surface integrin receptors responsible for interacting with the extracellular matrix (ECM) is adjusted in the ECs to accommodate the changing microenvironment. The signaling induced by the engagement of ECM proteins and corresponding integrin receptors regulates endothelial cell functional phenotype. The critical role in atherogenesis and the accessibility of matrix/integrin engagement makes it an attractive therapeutic target for atherosclerosis. This minireview summarizes recent findings on the shear-induced ECM remodeling and integrin expression, and their roles in atherosclerosis (Figure 1).

## Basement Membrane

The basement membrane is the subendothelial ECM largely secreted by and closely interacting with ECs. The major components of the basement membrane in normal vessels include laminin and type IV collagen (Yurchenco and O’Rear, 1994). Both laminin and collagen IV promote homeostasis of ECs, and prevent disturbed flow-induced NF-κB activation and inflammation (Orr et al., 2005). The composition of basement membrane is regulated by hemodynamic patterns. Disturbed flow induces subendothelial matrix remodeling to a “provisional” matrix rich in fibronectin at atheroprone sites prior to other signs of atherosclerosis (Orr et al., 2005). The provisional ECM is initially named with regard to the transient ECM (enriched with fibronectin, vitronectin, and fibrinogen) that is deposited and subsequently cleared during wound healing (Stupack and Cheresh, 2002). A fibronectin-rich ECM primes ECs to activation by oxidized low-density lipoprotein (Yurdagul et al., 2014) and hyperglycemia (Green et al., 2014). Onset of atheroprotective laminar shear stress induces a transient fibronectin upregulation compared to static control *in vitro*, whereas atheroprone oscillatory shear stress initiates a steady increase in fibronectin expression through the activation of NF-κB. Furthermore, because fibronectin promotes NF-κB activation, disturbed flow creates positive feedback to sustain endothelial inflammation (Feaver et al., 2010). Whereas disturbed shear activates p21-activated kinase in ECs on fibronectin leading to enhanced NF-κB and inflammation, ECs on native basement proteins inhibits shear-induced p21-activated kinase activation through a protein kinase A-dependent pathway.

## Integrins

Integrins are transmembrane heterodimeric proteins consisting of α and β subunits, responsible for cell adhesion to ECM and transducing environmental cues and internal cell signals in both directions. There are at least 18 α subunits and eight β subunits through different combinations to form 24 distinct integrins (Hynes, 2002). ECs express a repertoire of integrins to mediate dynamic interactions between cells and ECM. Endothelium on a native ECM expresses the collagen-binding (α1β1 and α2β1) and laminin-binding (α3β1, α6β1, and α6β4) integrins for mediating EC anchorage within quiescent blood vessels. ECs on a provisional matrix express RGD-binding integrins (including α5β1 and αvβ3), resulting in EC activation for angiogenesis or in pathologic conditions (Stupack and Cheresh, 2002). αvβ3 critically mediates EC interactions with provisional matrix proteins and disturbed flow-induced pro-inflammatory signaling (NF-κB and p21-activated kinase activation) in early atherogenic stages. However, inhibiting αvβ3 does not affect all the shear-induced signaling, as Akt, endothelial nitric oxide synthase, and extracellular regulated kinase remain responsive (Chen et al., 2015). Endothelial α5β1 binding with fibronectin under low oscillatory shear stress phosphorylates (activates) the cytosolic nonreceptor protein kinase c-Abl, which then induces tyrosine phosphorylation (at Y^357^) and nuclear translocation of yes-associated protein (YAP), leading to pro-atherogenic gene expression and EC activation. The phosphorylation of c-Abl and YAP^Y357^ is significantly higher in atherosclerotic plaques of *Apoe*^−/−^ mice and in patients (Li et al., 2019). Of note, YAP can also be serine phosphorylated at S127 (for 14-3-3 binding and cytoplasmic retention) and at S381 (for ubiquitination and degradation), thus inactivated by the Hippo pathway (Yu et al., 2015). As such, unidirectional shear flow sustains the cytoplasmic levels of YAP^S127,S381^ phosphorylation and suppression in ECs for atheroprotection through RhoA inhibition (Wang et al., 2016b). Endothelial α5β1 upon binding with fibronectin also recruits phosphodiesterase 4D5 at adhesion sites, which subsequently connects with phosphatase PP2A and its regulatory subunit B55α. Fibronectin-induced PP2A holoenzyme assembly triggers YAP^S127^-dephosphorylation and facilitates YAP activation (Yun et al., 2019). Although no direct evidence, increased levels of YAP by disturbed flow in ECs is consistent with the possibility of YAP^S381^-dephosphorylation (lower ubiquitination and degradation; Wang et al., 2016a). Furthermore, to effectively transduce outside-in signaling, inactive integrins require inside-out signaling to make a conformational change and promote their binding with permissive ligands. Disturbed flow activates endothelial α5β1 by increasing calcium influx through the cation channel Piezo1, which activates PTP1B-dependent dephosphorylation of annexin A2. Annexin A2 then binds to integrin α5 and facilitates translocation to lipid rafts to induce integrin activation and ligation (Zhang et al., 2020). Integrin α5β1 activation is essential for fibronectin deposition and proinflammatory responses by atheroprone shear or oxidized low-density-lipoproteins (Yurdagul et al., 2014; Al-Yafeai et al., 2018). Interestingly, laminin-binding integrin α6β1 is also induced by disturbed flow (Hsu et al., 2019). α6β1 binds to other ECM molecules in addition to laminin, which will be discussed below.

## Matricellular Proteins

Matricellular proteins are a group of extracellular proteins that do not directly serve structural roles, but rather function contextually as regulators of cell-matrix interactions and cell function (Bornstein and Sage, 2002). A number of matricellular proteins have been implicated in atherosclerosis. Among them, CCN1/CYR61 is the most well-characterized example of how matricellular proteins may regulate the formation of atherosclerosis. CCN1, a member of the CCN matricellular protein family, binds to at least seven integrin receptors, including α6β1 and αvβ3 on ECs (Kireeva et al., 1998; Leu et al., 2003; Chen and Lau, 2009), thus regulating diverse cellular activities including cell adhesion, migration, differentiation, proliferation, and survival/apoptosis/senescence (Lau, 2016). CCN1 is expressed in atherosclerotic lesions in patients (Hilfiker et al., 2002) and in the *Apoe*^−/−^ mouse models (Hsu et al., 2019). CCN1 expression is upregulated by oscillatory shear stress, and downregulated by laminar shear stress in ECs (Wang et al., 2016a,b; Hsu et al., 2019). Shear-induced CCN1 binds to α6β1 and causes atheroprone phenotypic changes in EC *via* NF-κB activation, which further increases the expression of CCN1, α6, and β1 in a vicious circle. A peptide antagonist selectively targeting CCN1-α6β1 engagement has been tested and effectively inhibits disturbed flow-induced atherogenesis *in vitro* and *in vivo* (Hsu et al., 2019). α6β1 is expressed at basal levels in quiescent ECs and binds to basement membrane protein laminin at focal adhesion sites (Seano et al., 2014). A fibronectin-rich provisional matrix under disturbed flow releases α6β1 from focal adhesions (Chen et al., 2015). The combination of α6β1 upregulation and disassembly by disturbed flow results in more accessible receptor integrins for CCN1 action.

CCN2/connective tissue growth factor and CCN1 share similar integrin receptors and are co-expressed in advanced atherosclerotic lesions in mice (Schober et al., 2002). Both CCN1 and CCN2 are directly induced by YAP/TAZ in ECs under oscillatory shear stress (Wang et al., 2016a,b). CCN2 has been demonstrated to increase neointimal thickening in a rat carotid artery angioplasty model (Kundi et al., 2009). However, the role of CCN2 in atherosclerosis remains elusive.

Interestingly, the matricellular protein cartilage oligomeric matrix protein (COMP)/thrombospondin-5 inhibits disturbed flow-induced inflammatory EC activation and atherogenesis through inactivating integrin α5, thus preventing α5β1-fibronectin engagement (Lv et al., 2021). COMP deficiency leads to accelerated atherosclerosis, plaque calcification, and post injury restenosis (Wang et al., 2010). The degradation of COMP has been associated with atherosclerosis progression in patients with symptomatic carotid stenosis by measuring circulating levels of COMP and its fragments (Sandstedt et al., 2021). It is worth noting that COMP is mostly secreted by vascular smooth muscle cells in the arterial wall, not by ECs (Fu et al., 2016). It is likely that intimal smooth muscle cells are responsible for producing and distributing COMP protein to the basement membrane of endothelium.

## Discussion

Current therapy for atherosclerosis primarily targets hyperlipidemia, thrombosis, and more recently vascular inflammation (Libby and Hansson, 2018). No available treatments directly target the dysfunctional endothelium in atherosclerosis. Patients with the conditions, such as existing atherosclerotic plaque, in-stent restenosis, bypass graft occlusion, transplant vasculopathy, or aortic valve calcification, are prone to further development of atherosclerotic lesion due to endothelial dysfunction caused by disturbed blood flow in the affected vascular segments (Chiu and Chien, 2011). The interaction between ECM and endothelial integrin receptors becomes a favorable therapeutic target for its accessibility and its critical role in atherosclerosis discussed in this review. The caveat of blocking provisional matrix proteins, such as fibronectin, poses a threat of complications because of the diverse and indispensable functions of fibronectin (George et al., 1993; Liu et al., 2010). Alternatively, integrin α5 can be considered as a target. Though α5-null mutation causes embryonic lethality in mice (Yang et al., 1993), α5^+/−^ mice are viable and display significant resistance to disturbed flow-induced EC dysfunction and atherosclerosis (Sun et al., 2016), suggesting a benefit from lowering α5 activities. An integrin α5β1-blocking peptide (ATN161) successfully abolishes disturbed flow-induced YAP activation and atherogenesis in *Apoe*^−/−^ mice (Li et al., 2019). Additionally, CCN1-α6β1 engagement is proven a promising therapeutic target for atherosclerosis (Hsu et al., 2019). Because laminin-α6β1 signaling is important for endothelial homeostasis and function, targeting α6β1 may be at risk of losing the atheroprotective effect from the native ECM. The antagonistic peptide T1 (derived from an α6β1-binding sequence of CCN1) preferentially blocks α6β1 binding with CCN1, without affecting binding with laminin. T1 peptide effectively blocks flow-induced atheroprone activation in ECs and atherogenesis in mice (Hsu et al., 2019). Moreover, a COMP-derived peptidomimetics (CCPep24), designed as an agonist for the specific COMP-α5 interaction, provides atheroprotection against flow-induced EC activation and atherogenesis in mice (Lv et al., 2021). Together, synthetic peptides or peptidomimetics have been successfully used to offer selective inhibition on interactions between specific provisional matrix components and their integrin receptors, and validate the approach targeting the pairing between matrix proteins and integrins for atherosclerosis therapy.

## Author Contributions

The author confirms being the sole contributor of this work and has approved it for publication.

### Conflict of Interest

The author declares that the research was conducted in the absence of any commercial or financial relationships that could be construed as a potential conflict of interest.
